# Redox-Based Strategies against Infections by Eukaryotic Pathogens

**DOI:** 10.3390/genes14040778

**Published:** 2023-03-23

**Authors:** Cindy Vallières, Marie-Pierre Golinelli-Cohen, Olivier Guittet, Michel Lepoivre, Meng-Er Huang, Laurence Vernis

**Affiliations:** Institut de Chimie des Substances Naturelles, CNRS UPR 2301, Université Paris-Saclay, 91198 Gif-sur-Yvette, France

**Keywords:** Redox, therapeutic strategy, fungi, parasites

## Abstract

Redox homeostasis is an equilibrium between reducing and oxidizing reactions within cells. It is an essential, dynamic process, which allows proper cellular reactions and regulates biological responses. Unbalanced redox homeostasis is the hallmark of many diseases, including cancer or inflammatory responses, and can eventually lead to cell death. Specifically, disrupting redox balance, essentially by increasing pro-oxidative molecules and favouring hyperoxidation, is a smart strategy to eliminate cells and has been used for cancer treatment, for example. Selectivity between cancer and normal cells thus appears crucial to avoid toxicity as much as possible. Redox-based approaches are also employed in the case of infectious diseases to tackle the pathogens specifically, with limited impacts on host cells. In this review, we focus on recent advances in redox-based strategies to fight eukaryotic pathogens, especially fungi and eukaryotic parasites. We report molecules recently described for causing or being associated with compromising redox homeostasis in pathogens and discuss therapeutic possibilities.

## 1. Introduction, Scope, and Aims of This Review

### 1.1. Reactive Oxygen Species (ROS) and Redox System in Human Cells 

Oxygen-derived reactive chemical species or ROS are chemically reactive non-radical molecules involved in diverse biological functions. Under physiological conditions, ROS are important signaling molecules, and a balance between the generation and elimination of ROS ensures the proper function of ROS and redox-sensitive signaling proteins [1,2]. For example, a transient and rapid ROS burst is necessary for ROS-mediated signaling while excessive and prolonged ROS accumulation leads to oxidative stress that may disrupt the function of key transcription factors and signal-transduction pathways responsible for the development of various pathologies, including cardiovascular, neurodegenerative, inflammatory diseases, and cancer. 

**Figure 1:**
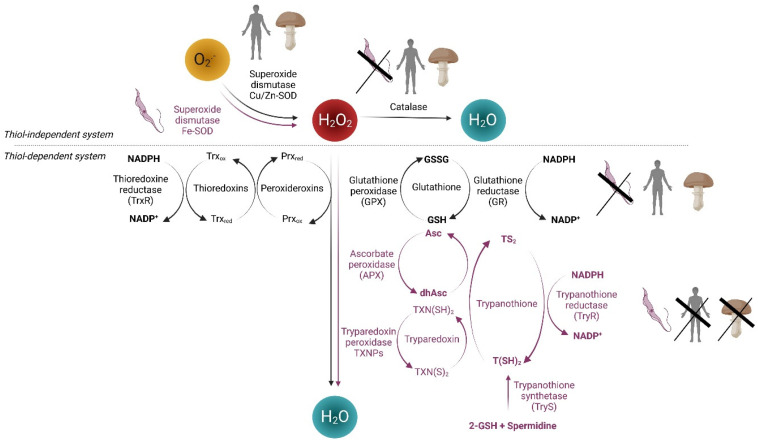
General scheme of redox systems in human, fungi, and parasites. Thiol-independent systems involve superoxide dismutases and catalases. Thiol-dependent systems are shown in black for human and fungi, mainly involving peroxiredoxins (Prxs) and thioredoxins (Trxs), on the one hand, and glutathione peroxidases (GPXs), on the other hand. Protozoa specificities are pictured in pink: two glutathione (GSH) molecules and spermidine are conjugated and catalysed by the trypanothione synthase. Trypanothione reductase (TryR) uses NADPH to maintain the trypanothione in its reduced form T(SH)_2_. In the cytosol, T(SH)_2_ reduces tryparedoxin TXN(S)_2_, which is will be used by the tryparedoxin peroxidases (TXNPs) to detoxify hydroperoxides (ROOH), and to deliver electrons to ribonucleotide reductase RNR, whose reduced form is required to produce dNTPs for DNA synthesis. T(SH)_2_ is also used for the conversion of dehydroascorbate to ascorbate by the ascorbate peroxidases (APXs), a reaction which is coupled to the reductive detoxification of H_2_O_2_.

Cellular oxidation-reduction (redox) systems are broadly defined networks that consist of ROS producers, antioxidant enzymes, and redox effectors as well as ROS and several small redox molecules (NADPH, glutathione, etc.) (Figure 1) [3]. Superoxide (O_2_•-) is the primary ROS produced from a variety of sources, including mitochondrial respiratory chain, NADPH oxidases, and a number of other biochemical reactions. Typically, molecular oxygen (O_2_) may react with electron generating superoxide that can be rapidly converted to hydrogen peroxide (H_2_O_2_) by superoxide dismutases (SOD). H_2_O_2_, the most important non-radical molecule, can be converted to water by catalases, peroxiredoxins (Prx), or glutathione peroxidases (GPX). In the presence of transition metals, H_2_O_2_ can be converted to hydroxyl radicals (HO•), which are highly reactive and can cause damage to all cellular components. Thiol-dependent antioxidants constitute the thioredoxin-based system composed of thioredoxin reductases (TrxR), thioredoxin (Trx), and Prx, and the glutathione-based system with several glutathione-utilizing enzymes, such as GPX, glutathione reductase (GR), and glutaredoxin. NADPH, which can be supplied by the pentose phosphate pathway, is the ultimate electron donor for both thioredoxin and glutathione systems. Finally, the downstream effectors of redox systems, which may be directly or indirectly redox-regulated, include transcription factors, such as nuclear factor erythroid 2-related factor 2 (NRF2), hypoxia-inducible factor 1-alpha (HIF1α), and nuclear factor NF-kappa-B.

Excessive oxidative stress leads to cell death. Thus, based on the difference in redox systems between pathogens and host (human), it is conceivable that pharmacological intervention through the generation of ROS and/or targeting redox systems could selectively kill or inhibit the pathogens with limited damage to host cells. Indeed, ROS generation and redox-based anticancer strategy are fields of increasing interest as cancer cells are generally in a persistent pro-oxidative status compared to their normal counterparts and are more vulnerable to further oxidative insults via pharmacological interventions [4,5]. In this review, we will first briefly describe our current knowledge on the distinct redox systems in several important pathogenic fungi and protozoan parasites; highlighting the features of the redox systems unique to these pathogens (Figure 1), and then, we present recent attempts at targeting redox systems directly or indirectly as a strategy against these disease-causing pathogens (Table 1). Indeed, both pathogenic fungi and parasitic protozoa remain severe threats to human health across the globe. Pathogenic fungi present a global threat for immunocompromised patients as well as for agriculture and global food security. Regarding parasitic protozoa, current chemotherapies may show several drawbacks, including usual lower efficiency in the chronic stage of these diseases, significant side effects, long treatment duration, and the existence of naturally or drug-induced resistant strains. In both cases, the adeptness of the pathogen and the emergence of resistant strains have further complicated the situation. Therefore, there is a pressing need to discover and develop alternative antifungal and anti-parasite drugs to combat such threats.

### 1.2. Redox Metabolism of Pathogenic Fungi 

Fungi share quite similar redox metabolism with human cells, making them hard to target using a redox-based therapeutic strategy. Fungi can infect multiple sites on the human body and cause both superficial and life-threatening infections. The most common human fungal infections come from several major fungal species, such as *Candida*, *Cryptococcus neoformans*, and *Aspergillus fumigatus* [6] (see also: *WHO fungal priority pathogens list to guide research, development and public health action.* World Health Organization. 2022. ISBN 978-92-4-006025-8). The host’s innate immune cells, including macrophages or neutrophils, are the first line of defense against fungal infections by phagocytizing and destroying the fungal cells via ROS generation. Like human cells, these pathogens possess the enzymatic (catalase, SOD, and peroxidase) and non-enzymatic (GSH) mechanisms to resist oxidative stress and ensure survival within the host. A robust ROS response capacity clearly contributes to fungal pathogenicity.

Fungal pathogens display differing degrees of sensitivity to the ROS and nitrogen species (RNS), yet they share high similarity in oxidative stress response mechanisms that protect them against these chemical insults [7,8]. Typically, fungal pathogens have conserved regulators central to such a stress response, (e.g., Hog1, Cap1, and Yap1), but there is divergence with respect to their upstream stress sensing mechanisms and some downstream transcriptional regulators. Increasing their sensitivity to host-generated ROS and RNS, sensitizing fungal pathogens to pharmacologically induced ROS and RNS or reducing their virulence and pathogenicity via redox manipulation are goals achievable by redox-based anti-fungi strategy.

### 1.3. Redox Metabolism in Protozoan Parasites

The major devastating protozoa-caused parasitic diseases are South American trypanosomiasis (Chagas disease), African trypanosomiasis (African sleeping sickness), malaria, and amoebiasis. Unicellular parasites of the trypanosomatidae family, responsible for Chagas diseases and sleeping sickness (Trypanosoma), respectively, as well as leishmaniasis (Leishmania), possess a redox system distinct from that of human cells [9]. These parasites heavily rely on the trypanothione, a thiol molecule that delivers electrons for the trypanothione-dependent pathways (Figure 1). Trypanothione contains two molecules of GSH joined by a spermidine linker, a reaction catalyzed by trypanothione synthetase [10]. It constitutes a central element in a peculiar trypanosomatid redox system, which includes trypanothione, a NADPH-dependent trypanothione-disulfide reductase, and a Trx-like protein called tryparedoxin, with a terminal electron acceptor, such as tryparedoxin-dependent peroxidase. Trypanothione reductase maintains trypanothione in the reduced state T(SH)_2_ using NADPH. Trypanothione directly reduces tryparedoxin, dehydroascorbate, and glutathione disulphide that, together with tryparedoxin peroxidase, ascorbate peroxidase, and GSH peroxidase-like enzymes, are responsible of the reductive detoxification of peroxides (H_2_O_2_, ROOH, and NOOH). The tryparedoxin plays an important role in catalyzing not only electron transfer from T(SH)_2_ to different molecular targets, such as peroxidases, but also ribonucleotide reductase and protein disulfides. Importantly, trypanosomatid cells lack catalase, GPX, and GR; therefore, their redox homeostasis is maintained mainly by biosynthesis, regeneration, and utilization of trypanothione, which represents a potential target for the development of new drugs to treat these diseases [9,11]. Indeed, many proteins of the parasite-specific trypanothione metabolism have shown to be essential for the survival of the parasites. 

*Plasmodium falciparum*, the cause of malaria in humans, is by far the most representative and important member of the Apicomplexa. The life cycle of *P. falciparum* involves an intracellular stage during which the parasites multiply and generate high levels of ROS inside the human erythrocyte. To protect its own survival and proliferation, *P. falciparum* relies on a well-equipped antioxidant system, including Trx- and glutathione-dependent proteins as well as SOD. In addition, *P. falciparum* possesses an exclusive redox-active protein called plasmoredoxin that is a member of the Trx superfamily. In particular, the *Plasmodium* species being deficient in catalase and glutathione peroxidase, the defense mechanism against peroxides relies on a set of five Prxs, differentially localized in the cytosol, mitochondria, apicoplast, and nucleus. Therefore, ROS induction and targeting proteins involved in antioxidant defense in malaria parasites have potential for the development of antimalarial drugs [12]. Another characteristic in most species of the phylum Apicomplexa, including *P. falciparum,* is the presence of apicoplast, a plastid-like organelle. The apicoplast hosts the ferredoxin redox system, including a ferredoxin and its NADPH-dependent reductase, which supplies electrons to various metabolic pathways in this organelle. The ferredoxin redox system is essential for the parasite and with no human counterpart, making the apicoplast also an attractive target for novel and much-needed antimalarial drugs [13].

*Entamoeba histolytica*, human pathogen for amoebiasis, is a typical representative of unicellular parasites Amoebizoa with essentially anaerobic metabolism. During tissue invasion, *E. histolytica* is exposed to elevated concentrations of exogenous ROS. A functional thioredoxin redox system consisting of Trx, the low molecular weight TrxR (L-TrxR) variant, and Prx play a key role in the maintenance of redox balance [14]. In particular, *E. histolytica* completely lacks GSH, and L-cysteine represents the major intracellular low-molecular-mass thiol. In addition, to contend with H_2_O_2_, two specific oxidoreductases are involved and act in concert, the pyruvate-ferredoxin oxidoreductase and the NADPH-dependent rubredoxin reductase. Interestingly, metronidazole, the drug of choice currently used for invasive amoebiasis, is activated via action of redox mechanisms inside the parasite. 

## 2. Targeting Redox Specificities in Parasites

An ideal target is a protein that is essential for the proliferation of the parasites but absent in their hosts or at least showing significant structural differences with the host protein. However, finding specific inhibitors of those enzymes is not an easy task. Reactive oxygen species (ROS), including hydrogen peroxide, superoxide radical, and hydroxyl radical, are by-products of oxygen metabolism in all cells. Parasitic infection leads to increased oxidative stress in the hosts. For instance, ROS are generated within the red blood cells infected by *Plasmodium* as a result of haemoglobin degradation in the food vacuole of the pathogen. Moreover, ROS arise from the production of oxygen radicals and nitric oxide by the immune system of the host in response to the microbial infection. Therefore, the parasites require efficient defense mechanisms to protect themselves against oxidative damage, such as specific ROS-detoxifying enzymes, in the particular absence of catalase. Several evidence points to the modulation of the redox system of parasites as a therapeutic strategy that is more rational than the manipulation of the redox metabolism of their vertebrate hosts. As several of those enzymes are not present in the hosts or differ strongly from their functional counterparts (see Figure 1), they represent potential molecular targets for novel drug development. In this section, we will present several parasitic proteins that are considered as promising drug targets and chemical compounds inhibiting those proteins. Interestingly, several inhibitors that have been designed for diverse human disorders also disrupt the redox metabolism of protozoan and helminth parasites, an effect that opens new avenues for drug repurposing for the treatment of infectious diseases.

### 2.1. Targeting Fe-SOD 

Superoxide dismutases (SODs) are key components of antioxidant defence systems of *Trypanosoma cruzi* (responsible for Chagas disease), as they convert superoxide anions into H_2_O_2_ and oxygen. The overexpression of such enzymes in the parasite confers a protection against host-derived pro-oxidants. Fe-SOD is absent in mammals [15] and, therefore, can be promising molecular targets for drug development against the kinetoplastid. In an early study, phthalazine and polyamine macrocycles derivates, for instance, are effective against *T. cruzi* in vitro and in vivo in the acute and in the chronic phase of the infection and showed selective inhibitory effects on the Fe-SOD enzymes of the parasite in comparison with human CuZn-SOD [16]. 

In the parasite *Leishmania infantum* (causing leishmaniasis), attempts to remove the FeSOD-A allele proved unsuccessful, highly suggesting that this gene is essential [17]. Further, downregulating its expression caused the parasite to be more susceptible to oxidative stress and decreased its ability to maintain infection in macrophages, confirming the rationale for searching for FeSOD-A inhibitors to fight infections not only by *L. infantum* but also by *T. cruzi* [11]. 

Not many recent advances were identified in developing such inhibitors. Six compounds were identified based on tetradentate polyamines, with anti-*Leishmania* activity in vitro against *Leishmania* promastigotes, three of them proving to be specific of Fe-SOD as opposed to Cu- or Zn-SOD [18]. Additionally, several Mannich base-type compounds were first designed and tested in vitro and in vivo proving interesting capacity to bind Fe-SOD and good antichagasic activity in a murine model, both in the chronic and acute phases [19,20], making these compounds worthy of being considered for further clinical testing. 

### 2.2. Targeting Trypanothione 

Catalase is unexpectedly absent in some members of the Kinetoplastida and Apicomplexa, two groups including parasitic protists. Trypanosomatids and *Plasmodium* are also lacking glutathione peroxidases that are also capable of rapidly metabolising high levels of H_2_O_2_. Instead, kinetoplastids have developed a unique antioxidant defence system against hydroperoxides based on trypanothione as stated in the introduction section (see Figure 1). APXs are class I heme-containing enzymes that play an important role in the antioxidant defence *Leishmania sp*. and *T. cruzi*. TryR, TXNPs, and APXs being absent in the human host, those enzymes can thus be considered as rational drug targets in the treatment of kinetoplastid infections (for review see [11]). The ancient arsenical drug melarsoprol, although highly toxic, is critical for the treatment of sleeping sickness caused by *T. brucei*, especially when the parasite enters the central nervous system. Within the cells, the compound is converted to melarsen oxide, which irreversibly binds sulfhydryl groups in T(SH)_2_. The complex forms the stable adduct MelT, a TryR inhibitor, ultimately disrupting energy production in the parasite. However, melarsoprol treatment being associated to high host toxicity and naturally occurring drug resistance, new drugs are urgently needed [21].

Interestingly, a kinetic model was set up to predict the reduction flux involving trypanothione and its reductase TryR [22]. Perturbation introduced in the TryR reduction rate was the main determinant for perturbing the system, leading also to a decrease in trypanothione synthesis rate. Consequently, iron–sulfur metabolism proved also disturbed, likely due to free radicals’ production. This model suggests that developing highly potent and specific inhibitors of the TryR enzyme should be of interest in treating leishmaniasis and an infection caused by *Trypanosoma*.

Several compounds were picked up in ZINC, a free database of commercially available compounds for virtual screening on the basis of their docking scores on a tridimensional model of *Leishmania Mexicana* trypanothione reductase (LmTR), and evaluated against recombinant LmTR [23]. Compound ZINC12151998, an N-(6-quinolinemethyl)-3-pyrazole carboxamide, showed higher leishmanicidal activity than reference treatments, such as glucantime, and is less cytotoxic than amphotericin B [24,25]. Additionally, new amino naphtoquinone derivatives, derived from compounds with activity on *T. cruzi*, proved potent on the epimastigote and trypomastigote forms of *T. cruzi* strains, and docking analysis also showed a good interaction profile with the enzyme for one of them [26]. Finally, novel symmetrical triazole analogues were designed to specifically bind the unexplored hydrophobic region at the homodimeric interface in the *L. infantum* trypanothione disulfude reductase (LiTR), proving to be non-competitive, slow-binding inhibitors of LiTR, by dramatically disrupting LiTR dimerization. Remarkably, leishmanicidal activity was enhanced, and both extracellular and intracellular parasites in cell cultures were killed [27].

All those compounds are now in the course of development and provide a good basis for the development of new anti-Leishmania drugs, even more potent and less cytotoxic.

## 3. Targeting the Thioredoxin Reductase TrxR

As described in the introduction section, fungal and human redox systems share a relatively general common organization, as opposed to parasites. In most fungi and human cells, redox systems rely on the coordinate activity of catalase, glutathione, and thioredoxin reduction chains (see Figure 1). As opposed to fungi and human cells, the parasite *Plasmodium* lacks catalase and glutathione peroxidase, so that the remaining thioredoxin system consisting of the thioredoxin reductase (TrxR) and its substrate thioredoxin (Trx) is essential for maintaining redox homeostasis and antioxidant defense in the parasite. TrxR of *P. falciparum* shows structural differences with TrxR identified in other Apicomplexan parasites and in humans. Indeed, an extended insertion loop sets PfTrxR apart from its human counterpart. The loop of the parasite consists of nineteen residues while hTrxR contains only five, leading to differences in substrate binding and reduction [28]. PfTrx1 in the cytosol and PfTrx2 in the mitochondria are the substrates of PfTrxRs and are necessary electron donors in the thioredoxin systems. Knockout of TrxR has a lethal effect on *P. falciparum* demonstrating the importance of the thioredoxin systems for the survival of the parasite [29]. This is not surprising since TrxR acts as a central redox regulator controlling critical cellular functions, such as the reduction in Prx and of the ribonucleotide reductase, as well as the repair of oxidized proteins via the reduction in methionine sulfoxide.

Several years ago, TrxR was identified as a target of metronidazole and other 5-nitroimidazoles in *E. histolytica* [30], *Trichomonas vaginalis* [31], and *Giardia lamblia* [32]. In anaerobic/microaerophilic organisms, 5-nitroimidazoles are toxic, as a strong reductive environment is needed to reduce the nitro group of the compound. This further causes the cytotoxicity of the drug, as 5-nitroimidazoles can form covalent adducts with cysteines in TrxR and inhibit its reductase activity [30,31]. However, similarly to auranofin, 5-nitroimidazoles have a pleiotropic mechanism, i.e., they damage DNA and bind to non-protein thiols, such as cysteine disrupting the redox status of the parasite.

In the paragraphs below, we review several compounds quite recently either identified, repurposed, or revisited as inhibitors of TrxR both in parasites and fungi.

Auranofin (AUF) is an anti-inflammatory FDA-approved compound used to treat rheumatoid arthritis. It has been known for a long time to exhibit antibacterial activity [33,34,35] but recent repurposing of AUF for new indications also included broad antifungal activity against a variety of medically important species [36]. *C. neoformans* was effectively inhibited with a minimum inhibitory concentration (MIC) of 0.5 µg/mL to 2 µg/mL [37,38,39,40]. Related, an NIH-sponsored phase I trial to characterize the pharmacokinetics (PK) and safety of auranofin in healthy volunteers was performed, indicating that plasma concentrations of AUF high above the MIC values for auranofin towards *E. histolytica* and *G. intestinalis* were reached [41].

AUF was shown to reduce *Candida albicans* biofilm formation [39]. One target of AUF was proposed to be thioredoxin reductase (TrxR) in *E. histolytica* [42] and in bacteria [43], but others suggested TrxR was not the primary target [44,45]. In an attempt to identify AUF targets using chemogenomic profiling, the *C. albicans* strains haploinsufficient for Mia40 (*mia40*) or for Erv1 (*erv1*) proved highly sensitive to the drug. Together with Erv1, Mia40 controls a disulphide relay system leading to protein being imported in the mitochondrial intermembrane space. These data suggest that Mia40/Erv1 is a new target of AUF, as resistance to AUF is conferred by Erv1 overexpression in yeast, which strongly supports this finding [39]. AUF also showed toxic effects on a broad range of parasites, i.e., *P. falciparum* [46], *L. infantum* [47], *E. histolytica* [47], and others of public health significance. It was suggested that the monovalent gold released from the drug could bind to the redox-active dithiol group of TrxR, therefore disrupting the electron transport to the thioredoxin. Previous studies using trypanothione reductase and glutathione-thioredoxin reductase incubated with AUF showed that co-crystals were formed where gold is bound to reactive cysteines in both proteins [48], where the glutathione-thioredoxin reductase is a unique fusion of a glutaredoxin domain with a thioredoxin reductase domain in the parasite *Schistosoma mansoni*. Further molecular dynamics studies showed that bound AUF to catalytic cysteines actually remains stable in the binding pocket of thiol-reductases in *L. infantum* and *P. falciparum* [49]. However, a tenfold overexpression of TrxR in *G. lamblia* did not confer resistance to AUF [50]. In addition, the drug indiscriminately reacts with cysteine as cysteine supplementation decreased *G. lamblia* susceptibility to AUF [51]. Isolation and characterisation of AUF-resistant *Toxoplasma gondii* strains did not highlight resistance mutations in the thioredoxin reductase gene [52]. Altogether, this suggests that TrxR is not the molecular target of the drug or at least not the only target in parasites. Interestingly, the resistant parasites all carry several mutations in genes encoding redox-relevant proteins, such as superoxide dismutase (TgSOD2) and ribonucleotide reductase. The authors suggested either a high threshold in AUF resistance development or that the compound interferes with many cellular processes and therefore more than one target needs to be altered to confer resistance to the drug. Taking into consideration recent results obtained in fungi, it is a possibility that Erv1/Mia40 might also be targeted by AUF in parasites, but this has not yet been assessed to date. 

Artemisinin is a well-known antimalarial molecule discovered by Pr Y. Tu in the early 70s [53]. Artemisinins, together with fexinidazole and benzonidazole used against *T. brucei* and *T. cruzi,* respectively, and metronidazole used to treat infections caused by intestinal protozoans, are drugs that are activated by chemical reduction inside the pathogens but not in the hosts, thus killing selectively the parasites (for review see [54]). The endoperoxide bridge of artemisinin is cleaved by a single-electron transfer. The potential electron donor is the ferrous heme, the end-product of haemoglobin degradation occurring in the digestive vacuole of *Plasmodium.* This is supported by some evidence, including the fact that blood parasites that do not digest haemoglobin, as *T. brucei* are less susceptible to artemisinin than *P. falciparum* [55]. The drawback of artemisinin is the very short half-life in the human body, rendering the drug inadequate for single-dose treatment. Novel antimalarial peroxides known as ozonides, which include arterolane and artefenomel, can overcome the short half time of artemisinin and the production issue [56,57]. Both compounds are synthetic, contrary to artemisinin, which is extracted from the plant *Artemisia annua*. The cleavage of artemisinin compounds (ARTs) generates alkylating carbon-centered radicals that react with heme, causing an increase in ROS production, leading to the parasite death. Chemoproteomics analyses showed that peroxide antimalarials disproportionately alkylate proteins involved in redox homeostasis and that impaired redox processes are involved in the mode of action of these antimalarials [58]. Artemisinin resistance is rapidly spreading. The antioxidant system, especially Fe-SOD and TrxR, is enhanced in the resistant strains, thus highlighting that artemisinin resistance is associated to the parasite ability to manage oxidative stress. The lower depletion of GSH in the artemisinin-resistant strain under artemisinin exposure demonstrates the importance of the GSH system in this resistance [59]. Interestingly, the inhibitors of TrxR are effective against wild-type and drug-resistant parasites. The use of molecules that specifically inhibit GSH synthesis in combination with artemisinin could also be a viable approach in winning the war against malaria due to the antimalarial resistance.

Importantly, artemisinin shows also antifungal activity against several species of Aspergilli (*flavus* and *niger*) and against *C. albicans* and *C. Cryptococcus* [60,61,62]. Several studies indicated that the mitochondrial NADH deshydrogenases are direct targets of artemisinin in yeast, leading to a potential disruption of membranes and a further ROS generation in yeast [63] and in *A. fumigatus* [64]. The same effect appeared to occur also in malaria parasites, *Plasmodium sp.* [65]. Transcriptomic and proteomic data analysis of *A. fumigatus* indicated that an oxidative phosphorylation pathway, as well as an ergosterol synthesis pathway, is consequently misregulated upon exposure to artemisinin [64], suggesting again that oxidative stress is produced during treatment by artemisinin. Interestingly, a stabilizing mutation in the ferredoxin, known to be involved in the oxidative stress response, confers resistance to parasites treated with artemisinin, in accordance with a protective role of ferredoxin against artemisinin [66]. 

Plasmodione (3-[4-(trifluoromethyl)benzyl]-menadione) is a new antimalarial drug. As a redox-active compound, it impairs the redox balance of parasites, mainly through the generation of benzhydrol and benzoyl metabolites leading to cell death [67,68]. Plasmodione and metabolites are actually substrates of mitochondrial respiratory chain flavoprotein NADH-dehydrogenases, leading to ROS production. Consistently, cells with a decreased antioxidant capacity show an increased sensitivity to plasmodione [69]. 

## 4. Targeting the Fungal TCA Cycle to Perturb the Redox Balance

In parasites, such as *P. falciparum*, most enzymes of the tricarboxylic acid (TCA) cycle are dispensable in asexual blood stages, except fumarate hydratase (FH) and malate–quinine oxidoreductase (MQO) [70]. However, asexual parasites can survive without a functional TCA cycle. For this reason, targeting the TCA cycle in parasites does not appear a rational approach at the asexual stage, even though TCA is now regarded as a target to prevent the parasite transmission after gametogenesis [71].

Opposite, several molecules targeting TCA in fungi have been identified. Ethanolic extract of *Humulus lupulus* containing isoxanthohumol exhibited moderate antifungal activity against five tested phytopathogenic fungi in vitro but proved highly efficient in vivo against *Botrytis cinerea*, a broad range plant pathogen. Interestingly, the antifungal mechanism was connected to the carbohydrate metabolic process: The TCA cycle as well as respiration were inhibited upon exposure to isoxanthohumol, consequently preventing ATP production [72]. Consequently, membrane lipid peroxidation occurred together with an increased expression of redox enzymes, including SOD, catalase, APX, and GPX, and of H_2_O_2_ concentration. These data thus point to a redox-regulated process, but the precise molecular target(s) of isoxanthohumol within the TCA process was not described yet.

Methylaervine is a natural antifungal agent with redox balance disrupting capacity. It exhibits effective activity against *Fusarium solani* (half maximal effective concentration EC50 = 10.56 µM) [73]. Exposure to methylaervine significantly induced lipid peroxidation, as malondialdehyde, the main product of polyunsaturated fatty acid peroxidation, was found to be increased. In addition, activities of two major antioxidant enzymes, catalase and SOD, were also increased. Interestingly, metabolomics analysis revealed that two major pathways are impacted upon exposure to methylaervine: The TCA cycle and steroid biosynthesis pathway. The activity of malate dehydrogenase and of succinate dehydrogenase were changed, suggesting the targeting of those two enzymes by methylarvine, accordingly with docking simulation studies [73]. 

## 5. Targeting Fungi through ROS Production

Phenazines are dibenzo-annulated pyrazines, which are found in nature and are of bacterial origin. *Pseudomonas aeruginosa* produces several phenazines, including the recently described 5-methyl-phenazine-1-carboxylic acid (5MPCA), exhibiting antifungal activity towards pathogenic fungi, such as *Candida albicans* [74]. This compound was found to generate ROS in vivo, which preceded 5MPCA-induced fungal death, suggesting that ROS production contributes to killing.

Interestingly, generating oxidative stress has been found to exacerbate the antifungal activity of conventional drugs, namely amphotericin B and itraconazole (ITZ), two drugs targeting ergosterol in fungal membranes [75]. The same strategy proved also true as *Aspergillus fumigatus* cells could be chemosensitized to the antifungal drug bithionol, a potent inhibitor of soluble adenylyl cyclase, by redox-active natural compounds or structural derivatives, such as thymol, and derivatives 4-isopropyl-3-methylphenol or 3,5-dimethoxybenzaldehyde [76].

Numerous nanoparticles (NPs) are being commonly used as potent antifungal agents for food preservation and also to fight fungal infections in humans [77]. Antifungal properties can occur through several mechanisms, including membrane damage, interactions with DNA, ion release, damage to hyphae and spore, ROS generation, and impact on mitochondria [78]. In the frame of this review, we will focus on this last one. Many studies indicated that intracellular ROS production occurs in the presence of metallic NPs and that this production is influenced by the type and specific surface of nanoparticles [79]. NPs can generate ROS due to transition metals on their surface through Fenton or Haber–Weiss reactions [80]. During these reactions, hydrogen peroxide is reduced in the presence of transition metals (Fe^2+^, Cu^+^) to form a highly active and toxic hydroxyl radical, underlying the significant role of NPs in ROS-mediated cell damage and cell death [81]. Interestingly, NPs are not prone to induce resistance, as opposed to chemical drugs, but opposite, ROS generation is not so much cell-specific and may lack selectivity needed otherwise to combat fungal infections. 

Interestingly, new kinds of NPs, non-toxic chitosan-tripolyphosphate-chloroquine (CS-TPP CQ) nanoparticles, were developed that are biocompatible and biodegradable. They can both modulate the pro- and anti-inflammatory responses and trigger the redox-mediated parasite killing [82].

**Table 1:** List of molecules presented throughout this review.

Name of the Molecule	Target	Mode of Action	References
Amphotericin B	Fungal membrane	Ergosterol binding	[75]
Artemisinin	NADH dehydrogenases	Oxidative stress generation	[63,65,66]
Auranofin	Thioredoxin reductases; Mia40/Erv1 import relay	Anti-oxidative stress response pathway	[37,38,39,40]
Bithionol	Adenylyl cyclase	cAMP synthesis inhibition	[76]
5-methyl-phenazine-1-carboxylic acid (5MPCA)		ROS production	[74]
5-nitroimidazoles	Cysteines in TrxR	TrxR activity inhibition	[30,31]
Isoxanthohumol	TCA cycle and respiration	ATP production decreased, ROS production increased	[72]
Itraconazole	Fungal membrane	Ergosterol binding	[75]
Mannich base-type compounds	Fe-SOD	Fe-SOD inhibition	[19,20]
Melarsoprol	Pyruvate kinase enzyme		
Metallic nanoparticles		ROS production	[78,79,80,81]
Methylaervine	TCA cycle, steroid biosynthesis	Redox balance disruptuin	[73]
N-(6-quinolinemethyl)-3-pyrazole carboxamide	LmTR	LmTR inhibition	[23,26]
Plasmodione	NADH dehydrogenases	Oxidative stress generation	[68,69]
Tetradentate polyamines	Fe-SOD	Fe-SOD inhibition	[18]
Triazole analogues	LiTR	Slow-binding inhibitors of LiTryR	[27]

## 6. Conclusions

Effective treatments against eukaryotic pathogens are still lacking, mainly due to different obstacles, including the eukaryotic nature of both pathogens and hosts. Indeed, the close physiological proximity between host and pathogenic cells makes it difficult to find opportunistic therapeutic windows, as any treatment should be as harmless as possible for the host cells while killing efficiently the pathogens. Related, the identification of pathogen-specific targets is difficult. Some molecules have proven efficient by targeting specific proteins of the pathogens, but resistances logically appear rapidly. 

Redox homeostasis unbalance/modulation proved useful to amplify drugs’ effects, such as in cancer treatment, for example [83]. This is the rational underlying so called redox-based approaches, using pro-oxidant compounds exhibiting synergistic effects when administered together with conventional treatments. As considered in this review, many newly identified compounds with antifungal or anti-parasitic properties also possess pro-oxidant capacity. This feature suggests that increasing oxidative stress using pro-oxidative compounds, administered together with antifungal or anti-parasitic treatments, might be of therapeutic interest. This has already been assessed using a single molecule capable of both generating massive oxidative stress and enhancing chloroquine action with effects on both sensitive and resistant parasites [82]. Combining two molecules in a therapeutic perspective would thus appear appealing, making it easier to fine-tune redox modulation and avoid possibly diminished or altered immune response in host cells due to hardly controlled redox perturbation.

## Data Availability

Not applicable.
